# Spinal metastasis of glioblastoma multiforme before gliosarcomatous transformation: a case report

**DOI:** 10.1186/s12883-020-01768-3

**Published:** 2020-05-11

**Authors:** Bing-Hung Hsu, Wei-Hwa Lee, Shun-Tai Yang, Cheng-Ta Han, Yuan-Yun Tseng

**Affiliations:** 1 Division of Neurosurgery, Department of Surgery, Shuang Ho Hospital, Taipei Medical University, No. 291, Zhongzheng Rd., Zhonghe District, New Taipei City, 235 Taiwan, R.O.C.; 2 Department of Pathology, Shuang Ho Hospital, Taipei Medical University, Taipei, Taiwan; 3 Department of Surgery, School of Medicine, College of Medicine, Taipei Medical University, Taipei, Taiwan

**Keywords:** Glioblastoma multiforme, Secondary gliosarcoma, Proton beam therapy, Spinal metastasis

## Abstract

**Background:**

Glioblastoma multiforme (GBM) is one of the most aggressive malignant brain tumors. Intracranial GBM metastases to the spine are rarely detected clinically. Secondary gliosarcomas after treatment of primary GBM are rarely described.

**Case presentation:**

Herein, we report the case of a 53-year-old woman who presented to our emergency room with progressive headache and weakness on the left side. Plain computed tomography and contrast magnetic resonance imaging of the brain revealed an approximately 6.8 cm × 4.5 cm right temporoparietooccipital intraaxial cystic tumor with surrounding diffuse perifocal edema that caused midline shift toward the left. Emergency craniotomy was performed to remove the tumor, and pathological examination revealed GBM. The patient received proton beam therapy, Gliadel implantation, and oral temozolomide chemotherapy as well as targeted therapy with bevacizumab. Approximately 15 months after diagnosis, she underwent surgical resection of the right temporal recurrent tumor and was newly diagnosed as having a metastatic spinal tumor. Pathologically, the right temporal and metastatic spinal tumors were gliosarcoma and GBM, respectively.

**Conclusions:**

Concurrent spinal metastasis and gliosarcomatous transformation, which are two types of GBM complications, are rare. To our knowledge, this is the first report of a case of recurrent GBM with gliosarcoma after proton bean therapy.

## Background

Glioblastoma multiforme (GBM) recurs locally and can extend along any stable fiber pathway, such as that from the anterior commissure to the optic radiations, the corpus callosum, and finally the fornix or cerebrospinal fluid (CSF) pathway. However, symptomatic spinal metastasis from intracranial GBM, although rare, may occur after clinical treatment of GBM [1–3]. Gliosarcoma (GS) is a rare GBM type composed of separate gliomatous and sarcomatous components [4, 5]. GS accounts for 1.8–8% of GBM cases and 0.48% of all intracranial tumor cases [6, 7]. Most types of GS are de novo and are thus termed “primary GS,” and those preceded by operative resection, radiotherapy, or chemotherapy are termed “secondary GS” [6–8].

Herein, we report the case of a female patient with a right temporal GBM. After surgical removal of the GBM, the patient underwent Gliadel wafer implantation, proton beam therapy, oral chemotherapy with temozolomide, and targeted therapy with bevacizumab. The GBM recurred as GS in the right temporal region and involved spinal metastasis in the T11–L1 region.

## Case presentation

**Fig. 1:**
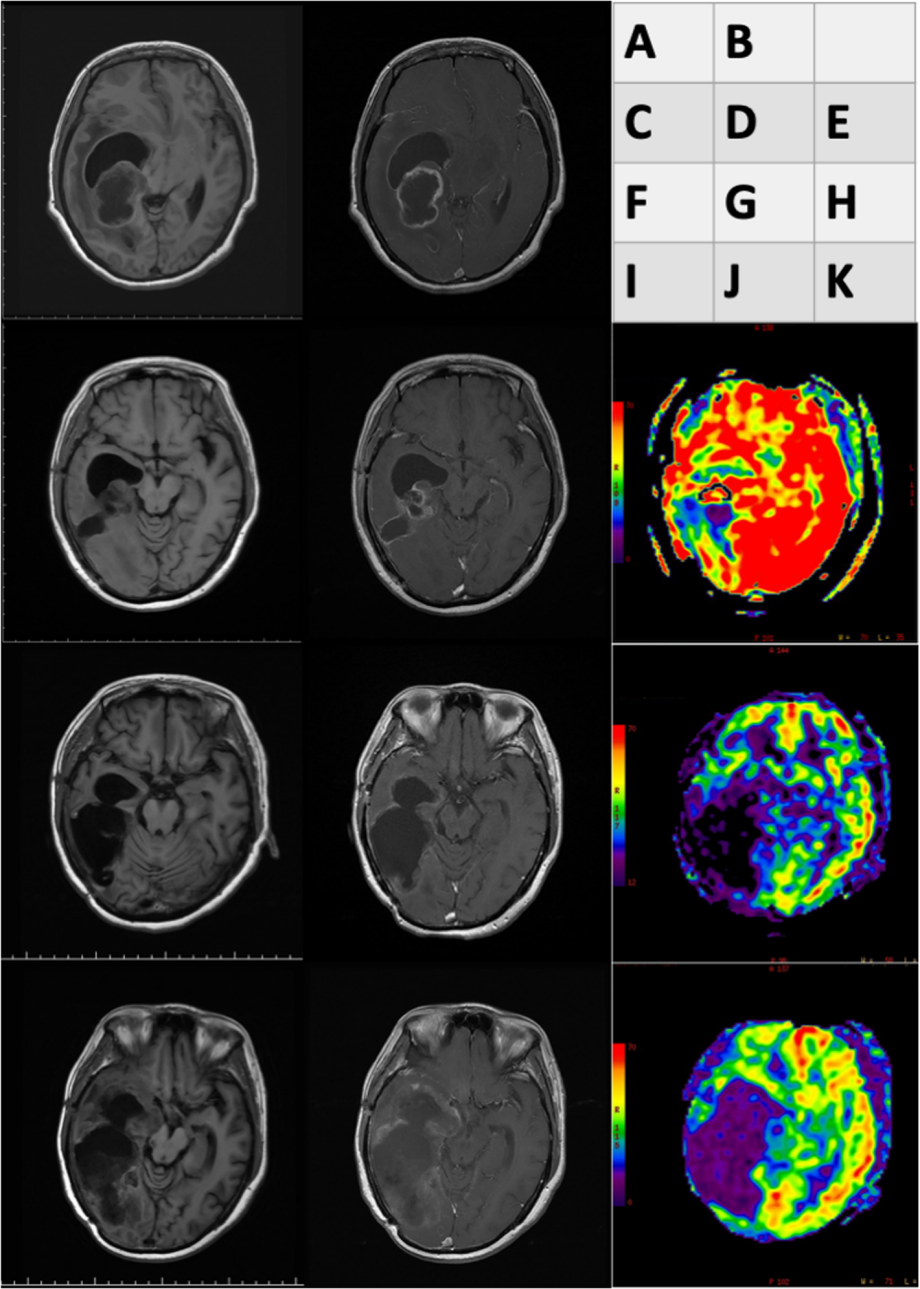
Brain magnetic resonance imaging (MRI) results for glioblastoma multiforme (GBM) that recurred at the same location as gliosarcoma (GS) after treatment. Initial T1-weighted (**a**) and postcontrast T1 FLAIR (**b**) brain MRI results obtained at the emergency department, demonstrating a large cystic mass with surrounding perifocal edema. T1-weighted (**c**) and postcontrast T1 FLAIR (**d**) and cerebral blood flow (CBF) (**e**) in the brain MRI after proton beam therapy, revealing mild interval enlargement of the right parietotemporal GBM with perifocal edema. T1-weighted (**f**) and postcontrast T1 FLAIR (**g**) and CBF (**h**) brain MRI after targeted therapy with bevacizumab, illustrating mild recurrence and avascular necrosis. Axial T1-weighted (**h**) and postcontrast T1 FLAIR (**i**) and CBF (**j**) image of secondary gliosarcoma. MRI revealed obvious tumor recurrence along the surgical margin. Tumor seeding was noted in the fourth ventricle, causing obstructive hydrocephalus and midbrain compression. The CBF indicated avascular necrosis

A 53-year-old woman presented to our emergency room with drowsiness and left-side hemiparesis. She complained of progressive headache and weakness on the left side within the past month. Plain computed tomography and contrast magnetic resonance imaging (MRI) of the brain demonstrated a nearly 6.8 cm × 4.5 cm right temporoparietooccipital intraaxial cystic tumor with surrounding diffuse perifocal edema that caused a midline shift toward the left and right lateral ventricle (Fig. 1a and b).

**Fig. 2:**
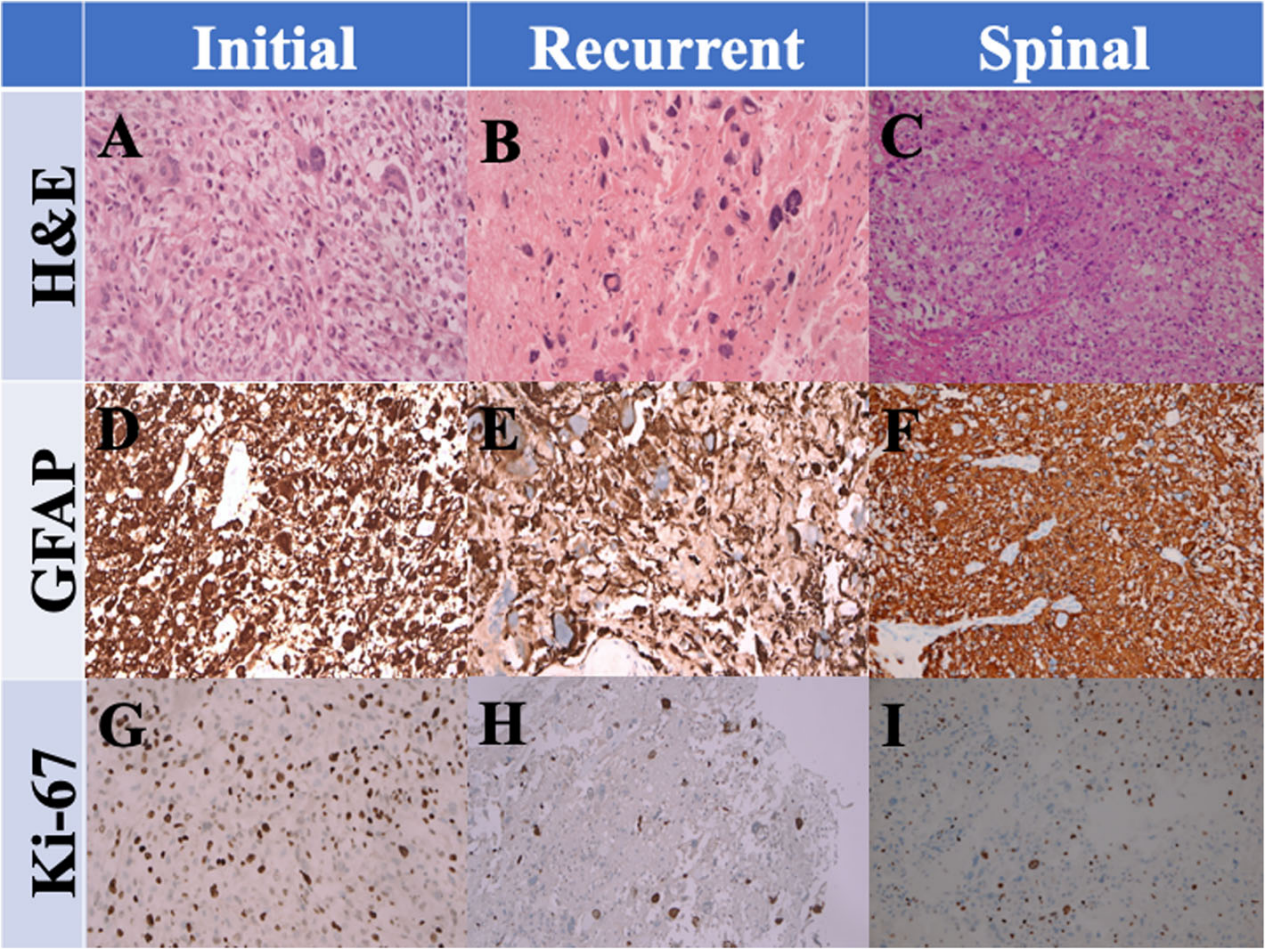
Photomicrographs of original brain and metastatic spinal glioblastoma multiforme (GBM). **a**–**c** Hematoxylin and eosin staining demonstrating high-grade glioma featuring nuclear atypia, cellular pleomorphism, mitotic activity, and remarkable necrosis. **d**–**f** The tumor cells were determined to exhibit glial fibrillary acidic protein (GFAP) positivity. **g**–**i** Ki-67 proliferative index was high (approximately 35%) initially; it was approximately 20% after proton beam radiotherapy and approximately 30% after spinal metastasis. Original magnification, 200×

For tumor removal, emergency craniotomy with decompression was performed. The extracted mass was soft and grayish, exhibited partial necrosis, and discharged yellowish fluid when cut piecemeal. The tumor mass was nearly entirely removed. A histopathological examination of the specimen revealed glioblastoma (World Health Organization grade IV) featuring nuclear atypia, cellular pleomorphism, mitotic activity, and considerable necrosis (Fig. 2a). Immunohistochemically, the tumor cells were focal positive for glial fibrillary acidic protein (GFAP; Fig. 2d) and negative for cytokeratin (CK) or S100. Staining for p53 and isocitrate dehydrogenase 1 (IDH1 R132H) revealed negative results. The Ki-67 proliferation index was approximately 30% (Fig. 2g).

**Fig. 3:**
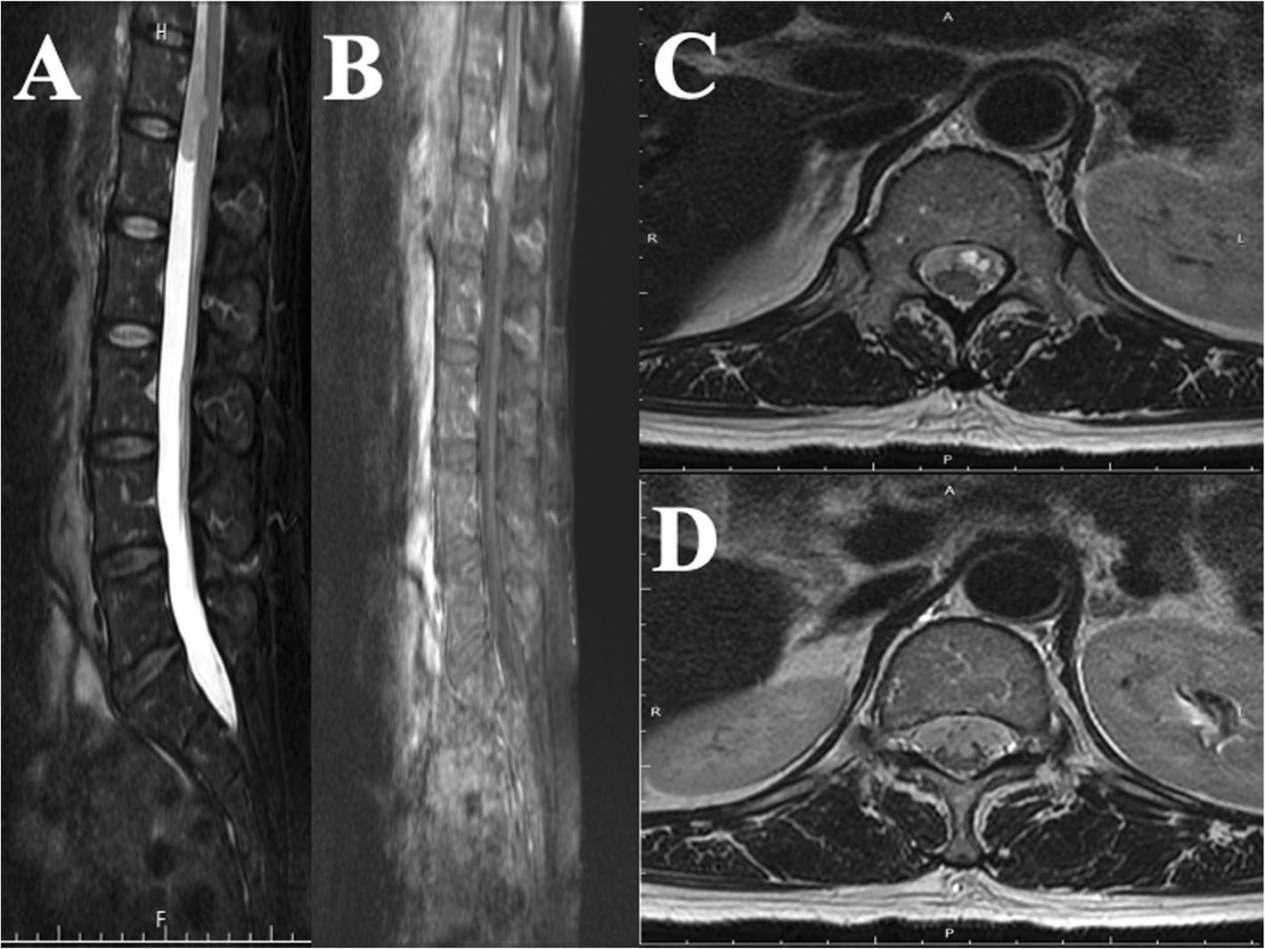
Spinal MRI results. **a** Sagittal T2-weighted image revealing a hypointense nodule at the T11–L1 level. **b** Postcontrast T1-weighted images depicting enhanced spinal tumor. **c** Axial section at the T12 level. **d** Axial section at the L1 level at the ventral aspect with conus medullary invasion

**Fig. 4:**
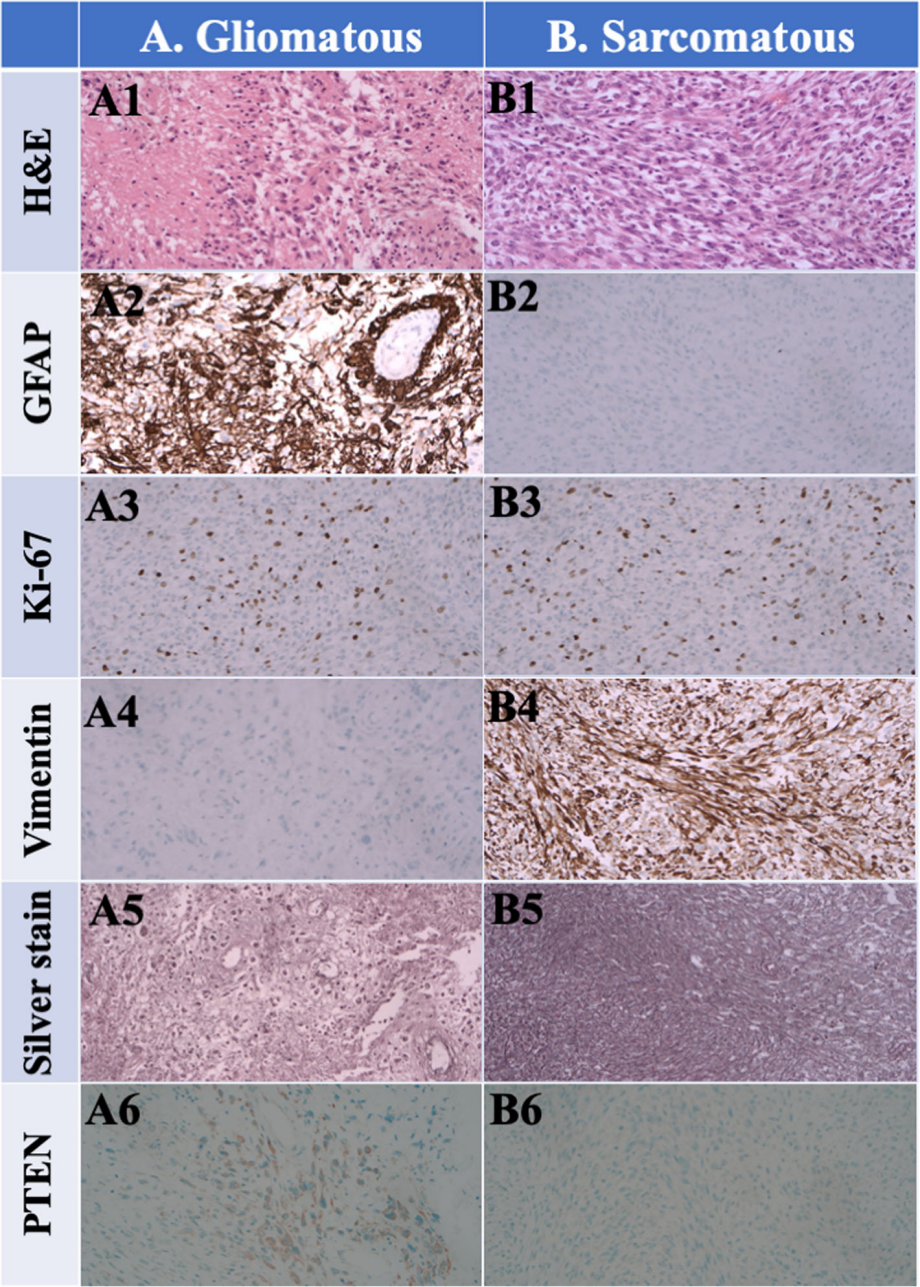
Gliosarcoma images. **a** Gliomatous component: hematoxylin and eosin staining (**a1**), glial fibrillary acidic protein (GFAP) positivity (**a2**), Ki-67 index (nearly 40%) (**a3**), vimentin negativity (**a4**), silver staining negativity (**a5**), and phosphatase and tensin homolo (PTEN) positivity (**a6**). **b** Sarcomatous component: hematoxylin and eosin staining (original magnification, 200×; **b1**), GFAP negativity (**b2**), diffuse staining with MIB-1 antibody (Ki-67 index > 50%) in tumor cells (i.e., abundant proliferative process), (**b3**), vimentin positivity (**b4**), silver staining positivity (**b5**), and PTEN negativity (**b6**). Magnification, × 200. (PTEN: phosphatase and tensin homolog)

After tumor removal, the patient recovered well and received a complete course of proton beam therapy (50.4 Gy in 28 fractions) in another medical center. After 3 months of therapy, MRI conducted during her regular follow-up (Fig. 1c, d, e) revealed a mild interval enlargement of the right temporoparietal GBM, measuring 4.2 cm × 3.5 cm at the largest diameter. For a more complete tumor resection, a second craniotomy for removing the recurrent GBM was performed under navigation assistance. A soft and necrotic mass with areas of rubbery consistency and gross appearance was extracted. After the surgical removal of the GBM, eight Gliadel wafers were implanted along the cavity walls and floor. Pathological reports indicated radiation necrosis and recurrent GBM with bizarre pleomorphic astrocytes (Fig. 2b)—which were positive for GFAP (Fig. 2e)—and with a high Ki-67 proliferation index (approximately 20%; Fig. 3h). The patient was administered a complete course of oral chemotherapy with temozolomide and targeted therapy with intravenously bevacizumab. The subsequent brain MRI revealed mild recurrent GBM at the right temporal lobe along the surgical margin (Fig. 1f, g, h). After 12 months, the patient presented with progressive headache, body weight loss, and easy fatigue. Under the suspicion of tumor recurrence, brain MRI was performed, which revealed obvious tumor recurrence along the surgical margin. Moreover, tumor seeding in the fourth ventricle caused obstructive hydrocephalus, midbrain compression, and midline shift to the left (Fig. 1i, j, k). A third craniotomy for removing the recurrent GBM was performed approximately 15 months after the GBM diagnosis. A histopathological examination of the extracted specimen revealed a bimorphic histopathological architecture, comprising gliomatous (approximately 30–35%) and sarcomatous (approximately 65–70%) components. The gliomatous component (Fig. 4a) consisted of high-grade glioma and considerable necrosis. Immunohistochemical studies demonstrated positivity for GFAP, a high Ki-67 proliferation index (approximately 40%), negativity for vimentin, negativity for silver staining, and positivity for phosphatase and tensin homolog (PTEN). The sarcomatous component (Fig. 4b) was composed of spindle-shaped sarcoma cells with increased cellularity, arranged in an interlacing pattern. Extensive necrosis was evident. Immunohistochemical examination revealed negativity for GFAP and IDH-1(wild-type), a high Ki-67 index (> 50%), positivity for vimentin, positivity for silver staining, and negativity for PTEN. The patient complained of urine incontinence, progressive and intractable back pain, and bilateral flank soreness approximately 1 month before this admission. Contrast L-spine MRI revealed an intradural tumor at T11–L1 level at the ventral aspect with conus medullary invasion (Fig. 3). Because of the patient’s urine/stool incontinence along with her unusual persistent back pain, T11–L1 laminectomy was performed to remove the intramedullary tumor 1 week after the third craniotomy. A soft and grayish tumor that bled easily and originated from the ventral side of the conus medullaris was noted. The metastatic spinal tumor was completely removed. Pathological examination confirmed glioblastoma with marked nuclear pleomorphism, microvascular proliferation, and focal necrosis (Fig. 2c). Immunohistochemically, the tumor cells were positive for GFAP (Fig. 2f) and negative for P53 and IDH-1. The Ki-67 index was approximately 30% (Fig. 2i). After the metastatic spinal tumor was removed, the intractable back pain was alleviated markedly, but the problem of urine/stool incontinence was not ameliorated. The patient died in January 2019, 20 months after the GBM diagnosis.

## Discussion and conclusions

The incidence of intracranial GBM metastasizing to the spine has been increasing in recent years [1, 9]. In a serial autopsy study, approximately 25% of patients with intracranial GBM demonstrated spinal subarachnoid seeding; however, the exact incidence remains unknown [2]. The incidence of GBM metastasizing to the spinal compartment is higher than that detected clinically, suggesting that such metastases have been underestimated thus far.

Symptomatic spinal dissemination develops in the later course of GBM; it is typically diagnosed 14 months after the initial diagnosis of brain GBM. Its treatment is palliative, and the prognosis is markedly poor; the average time interval between its diagnosis and death is 2 to 3 months [10]. These types of tumors may spread to the spinal subarachnoid space through direct extension, through the lymphatic system, from the bloodstream, and through the CSF. The involvement of the lateral or third ventricle (and the fourth ventricle in a few cases) is pivotal in the dissemination of CSF [1, 2, 11]. The most common spinal metastasis sites are the lower thoracic, upper lumbar, and lumbosacral regions [1, 12]. The initial brain MRI of the current patient revealed the involvement of the lateral ventricle and basal cistern. The patient was thus diagnosed as having intramedullary metastasis at T11–L1 approximately 15 months after cerebral GBM was confirmed.

GS is a rare histopathological variant of isocitrate dehydrogenase (IDH) wild-type GBM, and its corresponding median survival time after diagnosis is 6–14.8 months [5, 7, 13]. According to a literature review, secondary GS is extremely rare and has not been detected in patients with GBM. Even after multimodality treatment, including radical surgical resection, adjuvant irradiation, and chemotherapy, the prognosis of GS remains poor. The median survival time from the initial diagnosis GBM to the diagnosis of GS is 8.5 (range, 0.5–25) months [14, 15]. Previously reported clinical treatment–related experiences are limited. Currently, GS is treated using a protocol a GBM treatment protocol [8, 16]. Moreover, studies on the response of GS to advanced therapies typically used for GBM, such as immunotherapy, cancer vaccine therapy, and gene therapy, are limited. The prognosis of GS remains poor—poorer than that of GBM. The median survival time from GS diagnosis to death is 4.4 (range, 5.7–47.4) months [6, 17]. GS and GBM cannot be distinguished clinically; GS management strategies may be similar to those of GBM, with maximal safe surgical resection followed by chemotherapy and/or radiotherapy [16].

The causal relationship between therapeutic irradiation and delayed induction of neoplastic transformation is well established for meningioma, cerebral fibrosarcoma, and other sarcomatous variants; nevertheless, this relationship is not fully established for GBM. By studying a large case series of 32 patients with GS, Perry et al. [18] reported that only seven patients fulfilled the criteria for GS secondary to GBM. All these seven patients received 50-Gy whole-brain irradiation for the GBM. In the literature, most cases of secondary GS have been reported to be radiation-induced transformations [8, 18]. However, radiotherapy is not an absolute requirement for secondary GS. In a case series of 30 patients with secondary GS who underwent surgical resection for the management of the initial GBM, Han et al. [17] noted that 25 patients accepted external beam radiation combined with chemotherapy and that 3 underwent radiotherapy alone. The remaining two did not receive radiotherapy but demonstrated gliosarcomatous transformation: one patient was treated with chemotherapy alone, and the other underwent operative resection before the initiation of adjuvant therapy because of rapidly recurring GS. The patient received bevacizumab to treat the recurrent GBM, and no study has presented data demonstrating a causal relationship between sarcomatous transformation and bevacizumab therapy [8]. Proton beam therapy is an advanced-grade radiotherapy that precisely delivers high-dose radiation to a tumor site; thus, it is an excellent therapeutic choice for tumors located in the brain and other sensitive areas. Because positively charged ions can conform to the exact shape and depth of each tumor, the destruction of healthy surrounding tissue is minimized [19]. To our knowledge, this paper is the first to report the case of a patient with secondary GS after proton beam therapy.

Multiple reports have demonstrated that GS has a higher tendency to undergo metastasis compared with GBM (0.2–1.2%), and the tendency has been revealed to be as high as 11–15% in some studies [14, 15]. In our patient, secondary GS occurred after conventional adjuvant treatment was administered for GBM. Histopathological examination indicated that the spinal metastatic growth was GBM. Moreover, the spinal metastasis was pathologically similar to the initial GBM: positive for GFAP and vimentin and high Ki-67 index. The exact time of GBM cell dissemination to the spinal component remains unclear; however, we revealed that the GBM cells spread to the spine before the gliosarcomatous transformation occurred at the original tumor site.

In conclusion, we present the case of a patient who concurrently demonstrated two rare complications after undergoing standard treatment for GBM. First, gliosarcomatous transformation occurred at the original GBM site after surgical resection, interstitial chemotherapy with Gliadel wafers, oral chemotherapy with temozolomide, and proton beam therapy followed by targeted therapy with intravenous bevacizumab. Moreover, the intramedullary metastasis was from the GBM to the T11–L1 region.

## Data Availability

All data generated or analyzed during this study are included in this article.

## Abbreviations

GBM: Glioblastoma multiforme
CSF: Cerebrospinal fluid
GS: Gliosarcoma
CT: Computer tomography
MRI: Magnetic resonance imaging
GFAP: Glial fibrillary acidic protein
CK: Cytokeratin
IDH1: Isocitrate dehydrogenase 1
PTEN: phosphatase and tensin homolog

## References

[1] Birbilis TA, Matis GK, Eleftheriadis SG, Theodoropoulou EN, Sivridis E (2010). Spinal metastasis of glioblastoma multiforme: an uncommon suspect?. Spine (Phila Pa 1976).

[2] Erlich SS, Davis RL (1978). Spinal subarachnoid metastasis from primary intracranial glioblastoma multiforme. Cancer..

[3] Tseng YY, Kau YC, Liu SJ (2016). Advanced interstitial chemotherapy for treating malignant glioma. Expert Opin Drug Deliv.

[4] Kozak KR, Mahadevan A, Moody JS (2009). Adult gliosarcoma: epidemiology, natural history, and factors associated with outcome. Neuro-Oncology.

[5] Louis DN, Perry A, Reifenberger G, von Deimling A, Figarella-Branger D, Cavenee WK (2016). The 2016 World Health Organization classification of tumors of the central nervous system: a summary. Acta Neuropathol.

[6] Han SJ, Yang I, Tihan T, Chang SM, Parsa AT (2010). Secondary gliosarcoma: a review of clinical features and pathological diagnosis. J Neurosurg.

[7] Frandsen S, Broholm H, Larsen VA, Grunnet K, Moller S, Poulsen HS (2019). Clinical characteristics of Gliosarcoma and outcomes from standardized treatment relative to conventional Glioblastoma. Front Oncol.

[8] Andaloussi-Saghir K, Oukabli M, El Marjany M, Sifat H, Hadadi K, Mansouri H (2011). Secondary gliosarcoma after the treatment of primary glioblastoma multiforme. N Am J Med Sci.

[9] Lawton CD, Nagasawa DT, Yang I, Fessler RG, Smith ZA (2012). Leptomeningeal spinal metastases from glioblastoma multiforme: treatment and management of an uncommon manifestation of disease. J Neurosurg Spine.

[10] Vertosick FT, Selker RG (1990). Brain stem and spinal metastases of supratentorial glioblastoma multiforme: a clinical series. Neurosurgery..

[11] Lam CH, Cosgrove GR, Drislane FW, Sotrel A (1991). Spinal leptomeningeal metastasis from cerebral glioblastoma. Appearance on magnetic resonance imaging. Surg Neurol.

[12] Onda K, Tanaka R, Takeda N (1986). Spinal metastases of cerebral glioblastoma: the value of computed tomographic metrizamide myelography in the diagnosis. Surg Neurol.

[13] Guney Y, Hicsonmez A, Yilmaz S, Adas YG, Andrieu MN (2010). Gliosarcoma: a study of four cases. Rare Tumors.

[14] Salvati M, Caroli E, Raco A, Giangaspero F, Delfini R, Ferrante L (2005). Gliosarcomas: analysis of 11 cases do two subtypes exist?. J Neuro-Oncol.

[15] Dawar R, Fabiano AJ, Qiu J, Khushalani NI (2013). Secondary gliosarcoma with extra-cranial metastases: a report and review of the literature. Clin Neurol Neurosurg.

[16] Zhang G, Huang S, Zhang J, Wu Z, Lin S, Wang Y (2016). Clinical outcome of gliosarcoma compared with glioblastoma multiforme: a clinical study in Chinese patients. J Neuro-Oncol.

[17] Han SJ, Yang I, Otero JJ, Ahn BJ, Tihan T, McDermott MW (2010). Secondary gliosarcoma after diagnosis of glioblastoma: clinical experience with 30 consecutive patients. J Neurosurg.

[18] Perry JR, Ang LC, Bilbao JM, Muller PJ (1995). Clinicopathologic features of primary and postirradiation cerebral gliosarcoma. Cancer..

[19] Mizumoto M, Yamamoto T, Ishikawa E, Matsuda M, Takano S, Ishikawa H (2016). Proton beam therapy with concurrent chemotherapy for glioblastoma multiforme: comparison of nimustine hydrochloride and temozolomide. J Neuro-Oncol.

